# The Effects of Two Types of Sleep Deprivation on Visual Working Memory Capacity and Filtering Efficiency

**DOI:** 10.1371/journal.pone.0035653

**Published:** 2012-04-18

**Authors:** Sean P. A. Drummond, Dane E. Anderson, Laura D. Straus, Edward K. Vogel, Veronica B. Perez

**Affiliations:** 1 Psychology Service, VA San Diego Healthcare System, San Diego, California, United States of America; 2 Research Service, VA San Diego Healthcare System, San Diego, California, United States of America; 3 Department of Psychiatry, University of California San Diego, San Diego, California, United States of America; 4 San Diego State University and University of California San Diego (SDSU-UCSD) Joint Doctoral Program in Clinical Psychology, San Diego, California, United States of America; 5 Department of Psychology, University of Oregon, Eugene, Oregon, United States of America; 6 Department of Psychiatry, University of California San Francisco, San Francisco, California, United States of America; University of Pennsylvania School of Medicine, United States of America

## Abstract

Sleep deprivation has adverse consequences for a variety of cognitive functions. The exact effects of sleep deprivation, though, are dependent upon the cognitive process examined. Within working memory, for example, some component processes are more vulnerable to sleep deprivation than others. Additionally, the differential impacts on cognition of different types of sleep deprivation have not been well studied. The aim of this study was to examine the effects of one night of total sleep deprivation and 4 nights of partial sleep deprivation (4 hours in bed/night) on two components of visual working memory: capacity and filtering efficiency. Forty-four healthy young adults were randomly assigned to one of the two sleep deprivation conditions. All participants were studied: 1) in a well-rested condition (following 6 nights of 9 hours in bed/night); and 2) following sleep deprivation, in a counter-balanced order. Visual working memory testing consisted of two related tasks. The first measured visual working memory capacity and the second measured the ability to ignore distractor stimuli in a visual scene (filtering efficiency). Results showed neither type of sleep deprivation reduced visual working memory capacity. Partial sleep deprivation also generally did not change filtering efficiency. Total sleep deprivation, on the other hand, did impair performance in the filtering task. These results suggest components of visual working memory are differentially vulnerable to the effects of sleep deprivation, and different types of sleep deprivation impact visual working memory to different degrees. Such findings have implications for operational settings where individuals may need to perform with inadequate sleep and whose jobs involve receiving an array of visual information and discriminating the relevant from the irrelevant prior to making decisions or taking actions (e.g., baggage screeners, air traffic controllers, military personnel, health care providers).

## Introduction

Sleep deprivation can have a negative impact on many aspects of cognitive functioning. For example, sustained attention consistently shows performance impairment with sleep loss [1], [2], [3], [4]. Working memory, on the other hand, is a multi-component cognitive process for which impairment appears to vary depending on the exact component of working memory assessed [5], [6], [7], [8], [9], [10]. Broadly speaking, working memory can be differentiated into separate subsystems for verbal and visual information (e.g., [11]). While verbal working memory is fairly well studied in the context of sleep deprivation, relatively few studies have examined the effects of sleep deprivation on visual working memory.

Visual working memory (VWM) performance is thought to be comprised of two distinguishable component mechanisms. The first, capacity, involves the limits in the ability to simultaneously store and retain multiple pieces of visual information in working memory for short periods of time. Past studies testing VWM capacity have shown individuals are capable of retaining up to 3–4 objects at once, regardless of their complexity [12]. A second aspect of successful VWM involves controlling the flow of information into VWM, by determining whether stimuli are consistent with the individual's current goals. Since VWM capacity is limited, this process allows a person to “filter out” irrelevant information in order to focus on and remember relevant stimuli more efficiently [13]. Since working memory, in general, makes information available for more advanced cognitive processing, it represents one of the main rate limiting factors for higher-order cognitive functions such as fluid intelligence and complex decision making [14], [15], [16], [17], [18]. VWM may be particularly important in this fashion, as it is required for almost any cognitive demand involving storing multiple visual stimuli simultaneously or selecting target objects in crowded displays. Thus, to the extent the capacity or filtering components of VWM are impaired by sleep deprivation, this can have significant operational impacts for a wide range of individuals.

There has only been one study, to our knowledge, to examine the effect of sleep deprivation on VWM. Chee and Chuah [8] found one night of total sleep deprivation (TSD) significantly reduced VWM capacity, relative to after a normal night of sleep in the same individuals. They did not, however, examine the ability to identify relevant vs. irrelevant stimuli within VWM. Given the data showing different aspects of working memory can be differentially affected by sleep deprivation [5], it would appear crucial to examine both aspects of VWM to determine if one is more vulnerable to degradation than the other. Additionally, while some professions and/or individuals routinely experience the complete lack of sleep for one or more nights modeled by TSD, a larger number experience the type of sleep loss whereby individuals obtain some sleep, just an insufficient amount. This latter type of cumulative sleep loss, called partial sleep deprivation (PSD), may also affect VWM, but this has not yet been tested. In fact, few cognitive tasks have been examined under both TSD and PSD conditions and most of those have been in separate studies. Almost no studies have directly compared TSD and PSD, with the work of Van Dongen et al [4] the main exception. In that study, approximately 4–5 nights PSD (4 hours of sleep opportunity per night) produced deficits equivalent to one night of TSD for sustained attention, cognitive throughput, and subjective sleepiness.

The current study aimed to fill some of these gaps in our knowledge concerning the impact of sleep deprivation on VWM by examining two components of VWM (capacity and filtering) in the context of two types of sleep deprivation (TSD and PSD). To do this, we administered a computer-based test of VWM to healthy young adults after both six nights of sleep extension and either four nights of PSD or one night of TSD. The amounts of TSD and PSD were chosen, based on the limited data in the literature, in an effort to produce similar deficits in performance. Our VWM test contained two parts, designed to measure capacity and filtering efficiency, respectively, within VWM. We hypothesized: a) both sleep deprivation conditions would impair VWM capacity; b) both sleep deprivation conditions would impair filtering efficiency; and c) sleep deprivation would show a larger negative impact on filtering efficiency as the task becomes more difficult.

## Methods

### Ethics Statement

The study was approved by the ethics committees of the University of California San Diego and the VA San Diego Healthcare System. All participants provided written informed consent.

### Participants and Conditions

Forty-four healthy young adults were enrolled from the greater San Diego area to participate in this study (Table 1). Following written informed consent, all participants were screened for sleep disorders, drug use, psychiatric (Axis I) and medical disorders via structured interview and laboratory tests. Inclusion criteria included: a) age 18–39 years-old; b) regular sleep-wake schedule that included 7–9 hours time-in-bed with a bed time 2200-000 and a wake time 0600-0800; and c) no current and unmanaged medical or psychiatric diagnoses; and d) no personal history of any Axis I diagnosis or family history of mood or psychotic disorders. The sample size reported here came from a power analysis of the overall project's main aims, rather than from an estimated effect size on this specific task. The original power analysis called for 40 subjects. However, we enrolled 6 extra subjects to replace those subjects who had missing data related to the main study aims (which involved functional MRI of different cognitive tasks). Two of those subjects did not provide data for the current analysis for technical reasons (both underwent PSD first and WR second).

**Table 1:** Demographics.

Group	N	Gender	Age	Education
**TSD**	23	13F 10M	25.2±5.12	15.7±1.91
**PSD**	21	12F 9M	24.5±5.57	15.3±2.45
**Total**	44	25F 19M	24.9±5.29	15.5±2.17

PSD = partial sleep deprivation; 12 of these subjects underwent PSD prior to WR, 9 underwent WR prior to PSD.

TSD = total sleep deprivation; 10 of these subjects underwent TSD prior to WR, 13 underwent WR prior to TSD.

**Figure 1:**
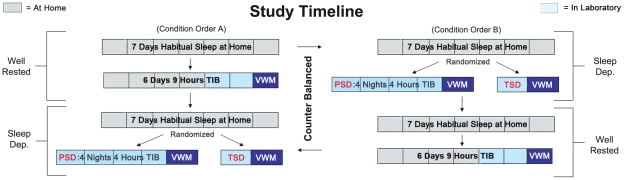
Protocol Timeline. There were two possible orders for the sleep conditions: either well-rested first or sleep deprivation first (order A and order B). Participants were randomly assigned to either the PSD or TSD groups and the order of sleep conditions was counter balanced across participants within each group.

All Participants were studied in two conditions: a Well-Rested (WR) condition and one of two randomly assigned Sleep Deprivation (SD) conditions (Figure 1): Partial Sleep Deprivation (PSD: four nights with four hours time-in-bed each night) or Total Sleep Deprivation (TSD: one night with no sleep). For the WR condition, participants spent six nights (four at home, two in the lab) with 9 hours time-in-bed each night to help ensure sleep satiation. Subjects were randomized in two stages. First, they were randomized to either the PSD or the TSD condition. Then, the order of WR and SD was counterbalanced across participants within SD condition. Thus, there were four possible experimental condition orders (WR-TSD, TSD-WR, PSD-WR, WR-PSD). Both WR and SD conditions were preceded by a 7-day period where participants maintained a regular sleep-wake schedule at home. This at-home sleep schedule was set to match each participant's self-reported habitual schedule as closely as possible. The center of that sleep period was used as the anchor for the WR and PSD conditions such that time-in-bed was extended or restricted, respectively, while maintaining the same center point. Adherence to the at-home sleep schedule was monitored via actigraphy, voicemail call-ins and diaries. Participants not fully adherent to their schedules were either rescheduled or dropped from the study. Test administration (see below) was scheduled based on each participant's wake time rather than clock time to help minimize differences in circadian phase during testing across participants. Sleep data from nights spent in the laboratory are reported in Table 2.

**Table 2:** Sleep Data from Nights in the Laboratory.

	WR night, TSD subj	WR night, PSD subj	PSD nights
**Total Sleep Time**	479.5±44.6 min	474.5±41.9 min	221.4±22.6 min
**Sleep Latency**	23.8±22.7 min	23.1±22.9 min	6.2±8.2 min
**Sleep Efficiency**	88.6±7.8%	87.6±7.7%	92.0±9.4%
**Stage 1%**	5.7±3.4%	7.0±4.1%	4.8±4.8%
**Stage %**	55±4.6%	52.7±7.0	39.1±10.9%
**Stage 3%**	15.9±5.0%	16.8±5.9%	35.1±11.3%
**REM%**	23.4±3.8%	23.5±4.9%	20.9±6.6%

WR = well rested; Time in Bed on the WR night was 9 hours. Data represents the 6^th^ consecutive night of 9 hours time-in-bed, which was the 2^nd^ night in the lab.

PSD = partial sleep deprivation = 4 hours time-in-bed per night. Data represents the overall average of the 4 nights of PSD in the lab prior to test administration.

While in the laboratory for SD, participants were constantly monitored in several ways. Sleep-wake state was monitored with actigraphy throughout the day and night, as well as polysomnography at night during the sleep period. Additionally, a 1∶1 staff∶participant ratio was maintained where research staff continually ensured wakefulness, while also documenting behavioral state, body position, activity, and vital signs at 15–20 minute intervals. Staff actively engaged with participants often and especially during times of overt sleepiness or circadian vulnerability to sleepiness. Participants were allowed to behave ad libitum when not undergoing testing, and were given access to games, books, TV, movies, and a computer with internet access. Participants were not allowed to leave the lab during the study and were restricted from caffeine and other stimulants while in lab. Prohibition of such stimulants, as well as alcohol, began 48 hours before the in-lab stays. Exercise and exposure to sunlight were also restricted beyond a daily 15 min supervised outdoor walk while wearing blue light-blocking sun glasses. This walk always occurred after the VWM test sessions.

### Task Procedure

Two VWM tasks utilized for this study were identical to those used by Vogel and colleagues [8], [13], [19], [20], [21], [22]. Each task assessed separate components of VWM: 1) capacity; and 2) filtering. The tasks were administered an average of 4.3±0.8 hours after waking during the well-rested condition (average clock time: 1213±46 min), 4.3±0.4 hours after waking following the fourth PSD night (average clock time 0939±27 min), and 22.3±0.5 hours into the TSD condition (average clock time 0604±29 min).

**Figure 2:**
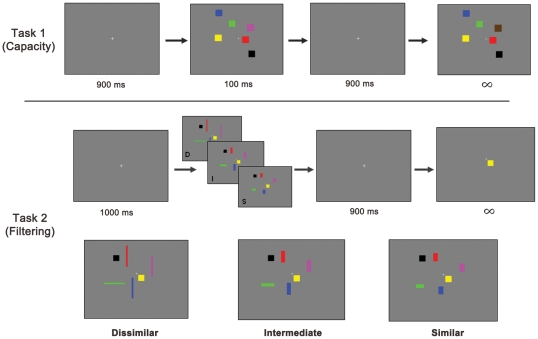
VWM Capacity and Filtering Tasks. These are examples of stimuli from each task. Participants always took Task 1 (VWM Capacity) before Task 2 (Filtering). Dissimilar, Intermediate and Similar distractors are each progressively more difficult to discern from target squares.

The task measuring capacity involved a target image composed of four to eight colored squares presented for 100 milliseconds (ms), followed by a fixation screen lasting 900 ms, and then a probe image with an identical number of squares as the original image (Figure 2). Participants were instructed to “remember as many of the colored squares as possible” from the first image, then identify whether the probe image was the same or different than the most recent target image. The instructions stressed accuracy over speed and, thus, the probe image was made available until the subject provided a response. The task consisted of 120 trials, equally distributed among target images containing 4, 6, or 8 squares. The outcome variable from this task was “K”, a behavioral measure of VWM capacity. VWM capacity is measured as **K = S (H−F)** where S is stimulus set size, H is the observed hit rate, and F is the false alarm rate [23], [24], [25].

The task measuring filtering assessed the ability to ignore irrelevant information and focus only on a subset of presented stimuli (Figure 2). This filtering task involved the same colored square stimuli as the capacity task. In this task, however, there were five separate types of trials. The first was identical to the 6-square version of the capacity task, except the probe image had only a single square and subjects were instructed to determine if it was “the same color and in the same location as in the target image.” This trial type was used as the baseline condition in determining filtering efficiency (see below). The second trial type contained two target squares, but was otherwise the same as the first. The final three trial types contained two target squares and four “distractor” rectangles, with each trial type representing a different difficulty level. Participants were instructed to “remember the squares and ignore all the rectangles”, and thus these trials required filtering irrelevant stimuli from the overall visual image. While total area was held constant across targets and distractors, the rectangular distractors varied in thickness and length such that they were relatively easy or progressively harder to discriminate from the target squares. There were three levels of distractors: “Similar”, “Intermediate”, and “Dissimilar”, with each level being increasingly different from the targets (Figure 2). As with the capacity task, probe stimuli in the filter task remained on the screen until the participant responded. There were 200 sets of images in the filter task, equally distributed among the five trial types. The main outcome variable of the task was a measure of filtering efficiency. Filtering efficiency is the increase in performance afforded by the ability to ignore the four distractor rectangles and focus VWM processes on only the two target squares. Therefore, if one is good at filtering out irrelevant distractors, then performance should be better in the filter conditions with only two targets, than in the baseline condition where there are 6 targets. Filtering efficiency was calculated separately for each filter condition and defined as **[Accuracy in each filter condition] – [Accuracy in the 6 square baseline condition]**. Thus, a filtering score equal to zero would reflect that performance for the “two targets plus four distractor” condition was the same as that for the “six targets” condition. Likewise, a filtering score greater than zero reflects a benefit of filtering relative to the six target baseline. Note some studies have instead calculated “filtering cost”, defined as the decrease in performance from the trials containing only two target squares with no distractors to the trials with distractors [26], [27]. Conceptually, the two contrasts are related, and we chose to focus on filtering efficiency in this study.

In addition to the VWM tasks, we administered the Psychomotor Vigilance Task (PVT) as a control task [28]. Since sleep deprivation, including both types employed here, is well known to produce deficits on the PVT, we administered this task as a manipulation check for our sleep deprivation conditions. We administered the PVT at 33 hours TSD and 12 hours after waking from the fourth night of PSD, both times representing about 5:00 PM. The task was programmed with Eprime software and administered on the same computer as the VWM tasks, but in all other ways it appeared visually identical to the original hand-held version of the PVT. The task begins with a blank box on the screen. At random intervals of 2–10 seconds, a millisecond counter begins to scroll within the box. The participants are instructed to hit a button to stop the counter as quickly as possible. Participants are given brief feedback on their reaction time and then the box goes blank during the next intertrial interval.

### Data Analysis

To examine VWM capacity (k), we ran a 2×2 (Group×Night) mixed model ANOVA (between subject Group = TSD vs PSD; within subject Night = WR vs sleep deprived). We planned to follow up a significant interaction by examining the main effect of Night for each Group. To examine filtering efficiency, we conducted a 2×2×3 (Group×Night×Difficulty) mixed model MANOVA (within subject Difficulty = Similar, Intermediate, Dissimilar distractors) where the primary contrast of interest was the 3-way interaction. If significant, we planned to follow up with a 2×3 (Night×Difficulty) repeated measures MANOVA for each Group separately. A significant interaction in those ANOVAs would be followed by paired-samples t-tests examining the effect of Night for each level of difficulty. To examine sustained attention via the PVT, we conducted a 2×2 (Group×Night) mixed model ANOVA on the number of lapses (reaction time >500 ms) and the median reaction time. Significant interactions were to be followed by the main effect of Night for each Group. We planned to examine main effects within any of these MANOVA where the interactions were not significant. Type I error was protected for each analysis at p = .05. Effect sizes are reported either as proportion of variance accounted for by a specific contrast of interest (partial r-squares) or as Cohen's d values [29].

## Results

**Figure 3:**
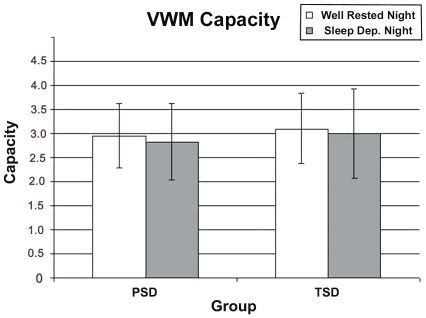
VWM Capacity. Values are mean ± standard deviation.

For VWM capacity, there were no statistically significant interactions or main effects within the 2×2 ANOVA (Figure 3 and Table 3). K scores were similar to those seen in the studies cited herein (WR mean across all subjects = 3.03, range = 1.57–4.37; TSD test session mean = 3.00, range = .63–4.23; PSD test session mean = 2.84, range = .93–4.00; more details in Table 3).

**Table 3:** Task Performance and Effect Sizes.

Task	Type/Level	Well Rested PSD		Sleep Dep. PSD		Effect Size PSD	Well Rested TSD		Sleep Dep. TSD		Effect Size TSD
		Mean	SD	Mean	SD	Cohen d	Mean	SD	Mean	SD	Cohen d
**Capacity**	K	2.953	±.668	2.835	±.801	−0.15	3.101	±.741	3.005	±.933	−0.15
**Filtering**	Dissimilar	.2212	±.094	.2275	±.092	0.06	.2336	±.086	.1861	±.101	−0.44*
	Intermediate	.2225	±.075	.1730	±.087	−0.50*	.2032	±.084	.1574	±.090	−0.44*
	Similar	.1662	±.096	.1560	±.103	−0.07	.1402	±.108	.0752	±.106	−0.52*
**PVT**	Median RT**	294.02	±37.7	309.93	±46.5	−0.43	287.98	±26.0	314.5	±43.8	−1.0
	Lapses**	1.85	±4.14	3.65	±5.10	−0.67	.69	±.97	2.52	±4.07	−1.1

Data shown are mean ± standard deviation. Effect sizes are Cohen d, with a negative effect sizes meaning worse performance with sleep deprivation. Data are shown separately for each Group on each Night.

* = p≤.05 for the simple main effect of Night between WR and sleep deprivation for the given difficulty level and sleep deprivation condition.

** = p≤.001 for the main effect of Night in the omnibus Night-by-Group ANOVA for the PVT measures.

**Figure 4:**
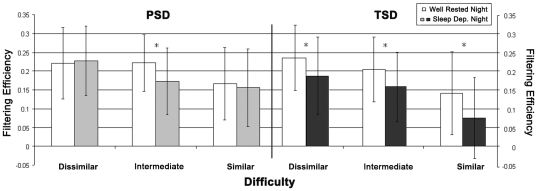
Filtering Efficiency. Filtering efficiency values are shown for both Groups, both Nights, and all 3 levels of Difficulty. Values are mean ± standard deviation. * = p≤.05 for the simple main effect of Night.

A significant 3-way interaction (p = .024; partial eta-square = .170) was found in the 2×2×3 (Group×Night×Difficulty) MANOVA focused on filtering efficiency (Figure 4 and Table 3). The follow up 2×3 (Night×Difficulty) repeated measures MANOVA for PSD showed a significant interaction (p = .008; partial eta-square = .414). The simple main effects of Night for PSD showed no change from WR to PSD for either the dissimilar or similar filtering trials but there was a significant decrease in filtering efficiency for the intermediate trials (Figure 4 and Table 3). For TSD, the follow-up Night×Difficulty MANOVA did not result in a significant interaction, but there were significant main effects of both Night (p = .018; partial eta-square = .228) and Difficulty (p<.001; partial eta-square = .634). During TSD, relative to WR, participants had a lower filtering efficiency for all three difficulty levels (Figure 4 and Table 3).

On the PVT, neither lapses nor median reaction time showed a Night×Group interaction or a main effect of Group. Both measures did show significant impairments with sleep deprivation (main effect of Night for lapses: p = .001; partial eta-square = .258; median RT: p<.001; partial eta-square = .285). See Table 3.

## Discussion

This is the first study, to our knowledge, to examine the effects of sleep deprivation on both VWM capacity and efficiency of filtering content within VWM. It is also the first to directly compare the impact of TSD and PSD on working memory of any kind within the same study. Sleep deprivation affected the components of VWM differently, and we found both similarities and differences across the types of sleep deprivation. Neither TSD nor PSD decreased VWM capacity. On the other hand, TSD reduced filtering efficiency at each level of difficulty while PSD generally did not affect filtering efficiency. Both types of sleep deprivation had the expected adverse impact on measures of sustained attention. These data suggest TSD can have a negative impact on our ability to ignore irrelevant stimuli and, thereby, avoid distraction in the visual environment.

One of our hypotheses, only partially supported here, was that TSD and PSD of the durations tested would show similar effects across different aspects of VWM. While the effect on VWM capacity was similar in the two types of sleep deprivation, the impact on filtering efficiency differed across types of sleep deprivation. Our hypothesis was based on the seminal and to our knowledge, only, paper directly comparing the two types of sleep deprivation [4]. However, our task relies on a different cognitive process than the measures presented in Von Dongen et al's paper sustained attention, cognitive throughput, and subjective sleepiness. This is critical given the growing body of data showing the exact effects of sleep loss are task and cognitive process specific (see below) and likely contributes to why we did not see equivalent effects across our two sleep deprivation conditions,.

### Cognitive Process-related Effects of Sleep Deprivation

Prior studies have shown sleep deprivation, especially TSD, can have differential impacts on different components of cognitive processing [4], [30], [31], [32]. Each of these studies demonstrated tasks assessing fundamentally different cognitive processes can show different levels of vulnerability to decline during TSD. Examining different aspects of a single cognitive process, Chee and Choo [7] reported maintenance and manipulation in verbal working memory are affected differently during TSD. Also examining verbal working memory, Turner et al [5] reported large deficits in capacity, moderate deficits in the gating function of working memory (related to, but not the same as, filtering) and no deficit in an index of encoding information from working memory after 42 hours TSD. These latter two studies suggest different components of the same cognitive process can be differentially affected by TSD. Our results extend that finding from *verbal* working memory to *visual* working memory, showing deficits in filtering efficiency, but not capacity, of VWM after one night TSD. This differential effect of TSD on different components of VWM also suggests TSD likely affects different brain regions to a different degree. While we did not directly assess brain function during VWM performance here, some hypotheses can be drawn based on related work.

### Potential Implications for Brain Function during Sleep Deprivation

Prior studies have shown storage of information in VWM during normally rested states relies on posterior visual processing regions such as visual association cortex and posterior parietal cortices [5], [25], [33], [34]. Our data suggest these regions can maintain sufficient responses following both one night TSD and 4 nights PSD to maintain normal VWM capacity. In the only other study to examine VWM during TSD, Chee and Chuah [34] administered a version of the capacity task we utilized here while subjects underwent functional MRI. They reported decreased VWM capacity after 24 hours TSD, attributing this to deteriorated perceptual processing and/or attention leading to a degraded representation of stimuli that is thus harder to maintain in working memory. Our findings are not consistent with those of Chee and Chuah, as we did not see a capacity decline. They may have seen greater deficits due to the sleep conducive functional MRI environment (dark, lying supine) making it harder for subjects to avoid microsleeps during TSD, thereby exaggerating effects on VWM. Due to our testing environment and task timing, we had less pressure for microsleeps and no missed responses. Nonetheless, the decline in filtering efficiency we found could still potentially be accounted for by a decline in perceptual processing and/or attention. We do not believe a decline in attention is responsible for our findings, though. While we have data showing the expected decline in sustained attention after TSD, such a decline was also seen after PSD, whereas filtering efficiency only decreased with TSD. On the other hand, if one night TSD had a greater impact on the ability to perceptually discriminate targets from distractors compared to four nights PSD, one would expect to observe reduced filtering efficiency like we did. This hypothesis is supported by the fact the effect size for the decline after TSD was (non-significantly) greater for the hardest filtering condition than for the two easier conditions, a finding consistent with a deficit in perceptual processing ability.

With respect to filtering efficiency, studies have shown a frontal-posterior network plays a role in successful performance in the presence of visual distraction. Specifically, Vogel et al [13] demonstrated capacity-related visual processing regions can discriminate those with high vs low filtering efficiency by demonstrating those with low filtering efficiency store distractor information over a delay period while those with greater filtering abilities do not. It is not clear from that study, though, whether these posterior regions represent the source of control over filtering efficiency, or if the signals measured represent the consequences of top-down control exerted from more anterior sites. Several authors, utilizing functional MRI, have identified dorsolateral prefrontal cortex (Broadmann's area 9/46) as an executive control region responsible for suppression of visual distraction [35], [36], [37], [38], [39], [40]. Dolcos et al [39], reported the inferior frontal gyrus may play an additional role the more similar a distractor is to target stimuli. These studies suggest frontal regions appear to play a gating function, selecting the information that is stored in posterior visual processing regions. Interestingly, several studies have shown those same prefrontal regions are sensitive to TSD, such that relatively intact performance on tasks requiring executive control is associated with increased activation following TSD, while impaired performance is associated with decreased activation [6], [7], [41], [42], [43]. Given that context, our pattern of results would suggest one night TSD, but not four nights PSD, impairs function within prefrontal regions responsible for determining relevant vs irrelevant information within VWM. However, once information passes to storage regions, the brain can maintain that information. Chee and Chuah's [8] findings would not necessarily support this latter implication. Overall then, the question regarding the source of cortical control over filtering efficiency, especially during sleep deprivation, remains unresolved, and serves as a ripe area future studies.

### Potential Operational Implications

Overall, we found one night of TSD impairs the ability to determine relevant vs irrelevant stimuli within a visual scene, while four nights of PSD restricted to 4 hours in bed/night does not. If replicated, this would have significant implications for operational settings. For example, airport baggage screeners constantly scan a multi-stimuli visual scene in an effort to determine the most relevant stimuli. Military personnel on patrol and in other operational environments must also observe complex visual scenes to determine relevant and irrelevant stimuli. Speculating if these findings are extended to other areas of working memory, they may apply to physicians trying to assess the importance of information coming simultaneously from patients, nurses, monitors, and other medical devices. One cautionary note is to not assume PSD has no deleterious effects on the ability to filter information in VWM. Although we did not find consistent effects on filtering efficiency during PSD, there is a hint of vulnerability during PSD with the findings in the intermediate distractor condition. More importantly, other types of PSD (e.g., increased number of nights or more restricted time in bed) may produce significant impairment in performance. Future studies should address this question.

### Limitations and Future Directions

A few limitations of this study should be acknowledged. First, we only studied one level of TSD and one level of PSD, and we only administered the task at one time of day. A greater understanding of the impact of sleep loss on VWM capacity and filtering would come from examining other lengths of TSD and PSD, as well as testing during other times of the day. Similarly, the TSD and PSD conditions differed on multiple dimensions, including homeostatic pressure, circadian timing (by 3–4 hours), proximal (i.e., the prior 4 nights) prior sleep history, and wake duration. These factors also inherently differ in real world settings when individuals are awake and performing under either total or partial sleep deprivation. This also argues for the need to extend the current research into other lengths of sleep deprivation and times of day to more fully understand the potential contributions of these various factors that necessarily co-occur and influence one another in the context of sleep deprivation. Third, the deficits we observed on the PVT, while significant, were not as large given the level of sleep deprivation as that reported in some other papers, suggesting our sample may have contained an unusual number of subjects relatively resilient to the effects of sleep deprivation on the PVT. We do not believe this fully explains our findings of no deficit on VWM capacity, though, for two reasons. Many studies focused on the PVT administer the task every 2 hours during the waking period and report the average performance across the entire day/night. In contrast, we administered the PVT at only a single time point that was not at an especially vulnerable time, from a circadian perspective (e.g., compared to the overnight period). In addition, studies have shown being resilient or vulnerable to performance deficits on the PVT, or any other measure, is not necessarily correlated with being resilient or vulnerable on other cognitive performance measures [5], [32]. So, it is hard to know if our subjects being resilient on the PVT would confer relative resilience on our VWM capacity measure. Furthermore, resilience on the PVT would not explain the differences we report between VWM capacity and flirting efficiency during sleep deprivation. Fourth, as with all sleep deprivation studies, we observed individual differences in performance following both TSD and PSD. Our sample size and study design did not allow for a priori examination of individual differences. However, a better understanding of such differences could potentially allow design of work schedules to minimize errors during periods of sleep deprivation or shift work. Similarly, developing and disseminating methods for training individuals to maintain filtering abilities during sleep loss may be beneficial, especially in operational settings. Finally, studies aimed at identifying neurophysiological mechanisms underlying deficits in VWM could also beneficial in designing counter measures to avoid those deficits.
